# UAV-based phenotyping outperforms visual canopy wilting for evaluating soybean drought tolerance and yield retention under rainfed conditions

**DOI:** 10.3389/fpls.2026.1835549

**Published:** 2026-06-02

**Authors:** Popat Shivaji Pawar, Chengjun Wu, Derrick Harrison, Daniel Rogers, Ben Fallen, Caio Canella Vieira

**Affiliations:** 1 Department of Crop, Soil, and Environmental Sciences, University of Arkansas, Fayetteville, AR, United States; 2 Nitrogen Fixation Unit, United States Department of Agriculture (USDA)-Agriculture Research Service, Raleigh, NC, United States

**Keywords:** canopy stress, drought tolerance, soybean breeding, UAV phenotyping, vegetation indices, yield retention

## Abstract

Drought is the major abiotic stress limiting soybean growth and yield, yet accurately identifying genotypes that sustain yield under rainfed conditions remains a major bottleneck in soybean breeding. Canopy wilting scores are widely used as a proxy for evaluating plant responses to drought stress. However, most assessments rely on leaf-level visual observations that are inherently subjective and typically based on single time-point scores, providing only a snapshot of stress expression and failing to capture their relationship with yield retention under rainfed conditions. To address these limitations, this study used Unmanned Aerial Vehicle (UAV)-based high-throughput phenotyping at a single growth stage (R4/R5) as a more quantitative and objective alternative to visual scoring, with closer relevance to yield performance under drought conditions. From 2023 to 2025, a total of 85 soybean genotypes developed by soybean breeding programs in Arkansas, Missouri, Kansas, and North Carolina, along with commercial checks, were evaluated under irrigated and rainfed conditions in Stuttgart, Arkansas. Visual canopy wilting scores were recorded at R4/R5, along with vegetation indices captured using UAV-based multispectral imagery. UAV-derived indices showed significant correlations with yield (*r* = 0.22 to 0.45, p<0.05) under rainfed conditions. In contrast, visual canopy wilting scores displayed weak and inconsistent associations with yield (*r* = -0.28 to 0.35, p<0.05), suggesting limited ability to capture yield retention under rainfed conditions. Unsupervised *k*-means clustering (*n* = 2) of UAV-derived vegetation indices separated genotypes into two distinct canopy response groups that were consistent across 2023 to 2025 rainfed seasons. Significant differences were observed among clusters for several vegetation indices (ARI, CIG, CIRE, GSAVI, GNDVI, GOSAVI, OSAVI, NDVI), indicating contrasting canopy stress responses. Under rainfed conditions, these UAV-defined clusters also differed for grain yield (2023: 1,925.6 *vs* 1,703.1 kg/ha; 2024: 1,849.9 *vs* 1,229.2 kg/ha; 2025: 2,056.7 *vs* 1,773.8 kg/ha), whereas visual wilting scores failed to distinguish yield-retaining genotypes. Overall, UAV-based high-throughput phenotyping offers a robust and yield-relevant alternative to visual wilting scores, supporting the development of drought-tolerant soybean germplasm and cultivars.

## 1 Introduction

Drought is the most critical environmental stress threatening the sustainability of global crop production, causing greater yield losses than any other abiotic stress (Boyer, 1982; Manavalan et al., 2009; Singh et al., 2022). Nearly 80% of global agricultural land is rainfed, placing approximately 60% of crop production at risk of drought-related yield reductions (Molden et al., 2011; Bao et al., 2019). Between 1983 and 2009, drought accounted for roughly 8% of global crop yield losses, corresponding to an estimated $166 billion in economic damage (Kim et al., 2019). In the United States, drought-related losses were estimated at about $14 billion in 2018 (Kuwayama, 2019). In recent decades, drought frequency and severity have intensified due to increasing population and food demand, limited water availability, and rising global temperatures (Dai, 2013; Trenberth et al., 2014; Malhi et al., 2021). Climate models project that this trend will continue with increasing drought events and associated agricultural losses in the coming decades (Singh et al., 2022; Parker et al., 2023; Thenveettil et al., 2025).

Soybean [*Glycine max* (L.) Merr.] is the world’s major oilseed crop and is highly susceptible to drought, resulting in substantial grain yield and quality losses across production regions (Irwin et al., 2017; Jin et al., 2017; Hamed et al., 2021; Soybean Research & Information Network, 2024). In the United States, more than 90% of soybean acreage is produced under rainfed conditions, leaving national production particularly exposed to water deficits (Jin et al., 2017; American Soybean Association, 2021). The 2012 drought alone reduced U.S. soybean production by more than 4.6 million metric tons, highlighting the scale of economic risk associated with drought-prone systems (Rippey, 2015).

Drought is a complex abiotic stress that affects soybean growth across all developmental stages, from germination to grain filling. Water deficits disrupt multiple morpho-physiological processes, including root development, photosynthesis, stomatal conductance, nutrient uptake, and reproductive success, ultimately reducing seed quality and yield (Fan et al., 2013; Vurukonda et al., 2016; Irwin et al., 2017; Bashir et al., 2019; Wijewardana et al., 2019; Du et al., 2020; Seleiman et al., 2021). Under rainfed production, soybean yield losses associated with drought range from 35% up to 80% depending on stress severity and genetic background (Specht et al., 2001; Guimarães-Dias et al., 2012; Wei et al., 2018; Marinho et al., 2022; Poudel et al., 2023). Importantly, yield reductions differ substantially between susceptible and drought-tolerant germplasm, emphasizing the value of identifying tolerant genetic resources for breeding (Sinclair et al., 2010; Devi et al., 2014).

Previous studies have identified key mechanisms associated with drought resilience, including slow canopy wilting, sustained nitrogen fixation, and reduced transpiration rates (Sloane et al., 1990; Vadez and Sinclair, 2002; Fletcher et al., 2007; Sinclair et al., 2008, 2010; Devi et al., 2014). These traits have been incorporated into breeding pipelines using drought-tolerant donor lines such as PI 416937, PI 471938, and Jackson, leading to the release of improved germplasm with enhanced yield stability under rainfed conditions, including USDA-N8002 and USDA-N7006 (Carter et al., 2016; Fallen et al., 2023), and elite Arkansas drought-tolerant lines such as R01-416F (Chen et al., 2007) and R19-42848 (Wu et al., 2025). Despite these advances, large-scale phenotypic evaluation of drought responses remains a major bottleneck because tolerance is expressed dynamically across the growing season and cannot be fully captured by a single trait or observation.

Traditional breeding programs rely heavily on manual visual scoring of drought-related traits. Visual wilting score is a field-based, manual assessment used to quantify drought-induced loss of leaf turgor in soybean. It is widely applied in soybean drought phenotyping studies conducted under water-limited field conditions (King et al., 2009; Hwang et al., 2015; Fallen et al., 2023). Although widely adopted, visual phenotyping is labor-intensive, low-throughput, and subject to observer bias, limiting scalability and repeatability in multi-environment field trials (Yang et al., 2017; Sankaran et al., 2018; Johansen et al., 2019; Fukano et al., 2021). Recent evidence further suggests that reduced wilting reflects drought-avoidance responses rather than consistent yield benefits, indicating that wilting severity alone does not provide a generalizable indicator of grain yield under rainfed conditions (Bellaloui et al., 2026). In contrast to visual wilting scoring, Unmanned Aerial Vehicle (UAV)-based high-throughput phenotyping provides a quantitative alternative by capturing canopy information across time and space using RGB and multispectral sensors. UAV platforms have been successfully applied to evaluate grain yield, biomass, plant architecture, and abiotic stress responses in multiple crops (Araus et al., 2018; Malambo et al., 2018; Gano et al., 2024). In soybean, spectral reflectance measurements have been shown to reliably capture plant water status and drought-induced physiological variation across growth stages and seasons, establishing a robust physiological basis for scaling vegetation indices to UAV-based drought phenotyping (Crusiol et al., 2023; Gonçalves de Paula et al., 2024). Consistent with this, UAV-derived vegetation indices have shown strong potential for assessing canopy condition and predicting agronomic performance under field conditions, with multispectral indices capturing water-stress-related structural and physiological responses (Maimaitijiang et al., 2020; Alabi et al., 2022; Tavares et al., 2022; Tahery et al., 2025). Recent UAV-based studies have strengthened soybean drought phenotyping by incorporating sensor-derived canopy metrics that capture stress-related variation under field conditions. For instance, Zhou et al. (2021) used a deep learning model with UAV imagery to predict soybean grain yield under drought stress; however, their analysis was limited to a single site and growing season, which constrains the assessment of consistency across environments. Liang et al. (2024) extended UAV phenotyping toward drought tolerance evaluation by incorporating yield-based resilience metrics, such as geometric mean productivity, using multimodal UAV data and machine learning. However, visual canopy wilting was not quantified, limiting direct comparison between UAV-based and wilting-based phenotyping. Burner et al. (2025) focused on characterizing drought-related canopy responses and their genetic basis, demonstrating strong associations between UAV-derived multispectral indices and visual canopy wilting, while evaluation of yield relevance was outside the scope of that study. Similarly, Jones et al. (2024) demonstrated that UAV-derived features can replicate breeder selections based on visual canopy wilting. Although yield correlations were reported to be weak, drought response was evaluated primarily through stress expression rather than through a yield-focused phenotyping framework. While these studies highlight the promise of UAV phenotyping, it remains unclear whether UAV−derived vegetation indices can outperform traditional canopy−wilting scores in consistency, predictive value, and practical utility for drought−tolerance selection under rainfed field conditions. This study aimed to compare UAV-based and visual drought phenotyping approaches, evaluate the effectiveness of UAV-derived vegetation indices for assessing soybean drought tolerance, quantify their relationships with yield, and develop an integrated phenotyping framework to improve the selection of soybean genotypes with higher yield retention under rainfed conditions.

## 2 Materials and methods

### 2.1 Plant materials

A total of 85 soybean advanced breeding lines (35 maturity group IV and 50 maturity group V) developed by public soybean breeding programs in Arkansas, Missouri, Kansas, and North Carolina were evaluated for drought tolerance (**Supplementary Table 1**). The trials included one conventional drought-tolerant check (Ellis) and high-yielding commercial checks (AG 4135, AG53X9, LD15-3818, Hutcheson). The breeding lines were derived from high-yielding, drought-tolerant pedigrees and had previously undergone multi-trait selection in preliminary and advanced yield trials within their respective state programs.

### 2.2 Field trials and visual data collection

**Figure 1:**
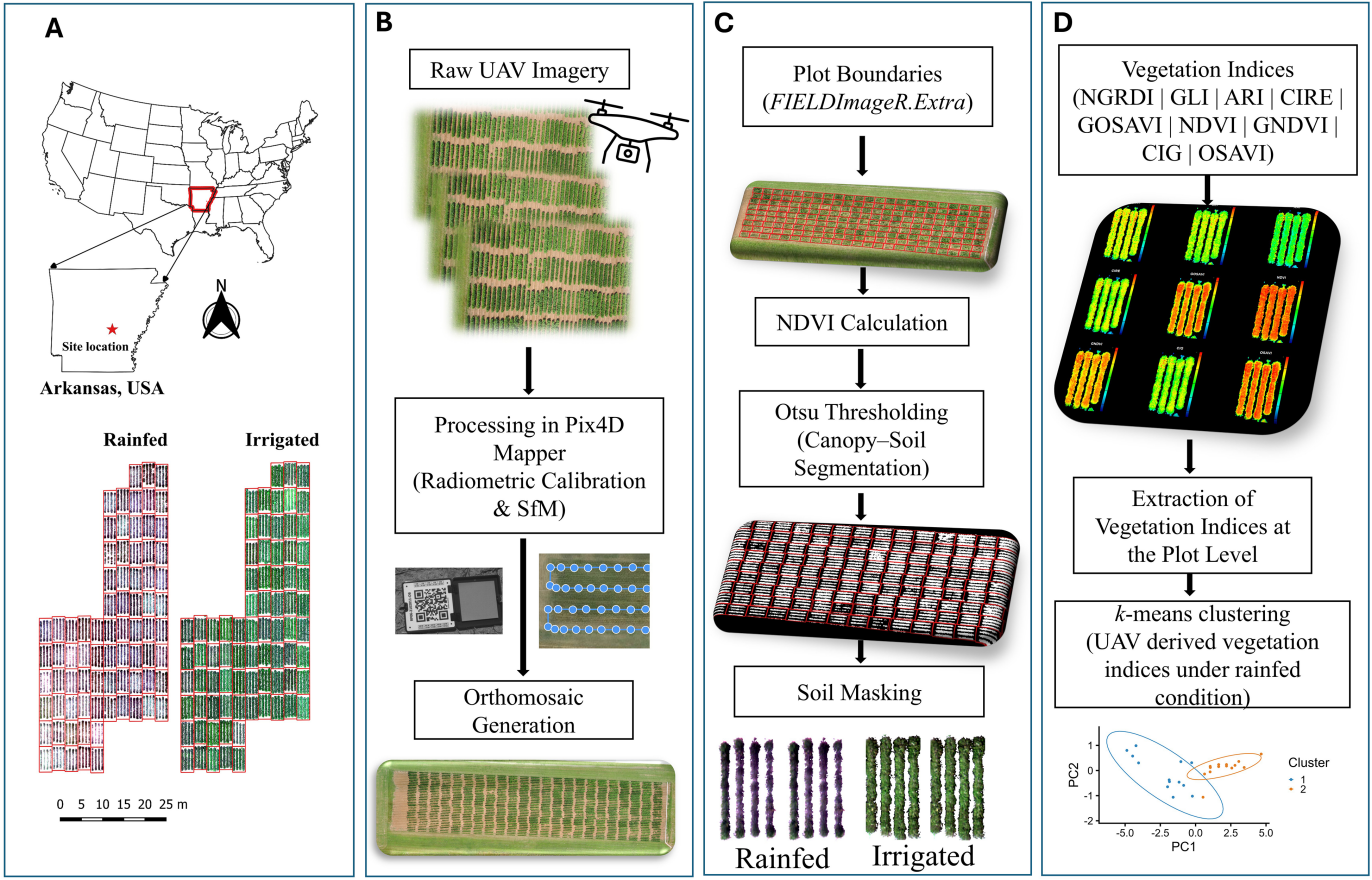
UAV-based high-throughput phenotyping workflow. **(A)** Study area at Rice Research & Extension Center (RREC), Stuttgart, Arkansas, showing USBDT field trial plots. **(B)** Raw UAV image acquisition and preprocessing. **(C)** Field plot boundary extraction and plot-level segmentation. **(D)** Vegetation index computation followed by *k*-means clustering of UAV-derived vegetation index data.

Field trials were conducted at the Rice Research & Extension Center (RREC) in Stuttgart, Arkansas (34°27’56.1” N, 91°25’20.4” W) in a DeWitt silt loam soil under side-by-side irrigated (IRR) and rainfed (DT) conditions (**Figure 1A**). Field plots consisted of four rows, each 4.57 m long with 0.76 m spacing. Entries were arranged in a three-replicate, randomized complete block design. Trials were planted on 10 May in 2023, 14 May in 2024, and 18 April in 2025 (**Table 1**).

**Table 1:** Field trial summary with entries, checks, and key dates.

Year	Entries	Maturity group	Checks	Planting date	UAV flight date	Wilting score date
2023	13	IV	LD15-3818, AG 4135	10 May	30 Aug	30 Aug
	19	V	Hutcheson, AG53X9, Ellis			
2024	10	IV	AG 4135, LD15-3818	14 May	13 Aug	13 Aug
	16	V	Hutcheson, AG53X9, Ellis			
2025	12	IV	AG 4135, LD15-3818	18 April	30 July	30 July
	15	V	Hutcheson, AG54XF5, Ellis			

Visual canopy wilting scores were collected from rainfed plots at the R4 (Full pod) to R5 (Beginning seed) growth stages (Fehr and Caviness, 1977), following at least 10 consecutive days without rainfall, when soil moisture was ≤ 0.15 m³/m³ and soil temperature was ≥ 35 °C. A single assessment was conducted within this interval, as soybean is highly sensitive to drought during this reproductive stage, which strongly affects seed development and yield (Farooq et al, 2017; Du et al., 2020). Environmental conditions were monitored using a ZL6 data logger and soil sensors connected to the ZENTRA Cloud platform (METER Group Inc., USA). Canopy wilting was rated on a 1–5 scale, where 1 = no visible wilting, 2 = slight wilting, 3 = moderate wilting, 4 = severe wilting with partial defoliation, and 5 = partial plant death (Carter et al., 2016; Fallen et al., 2023; Wu et al., 2025). Representative canopy images for the observed wilting score levels are provided in the **Supplementary Figure 1**. Grain yield from each plot was harvested using a plot combine and adjusted to 13% moisture content.

### 2.3 UAV image data collection

UAV-based imagery was collected during the soybean R4 to R5 growth stages between 2023 and 2025 on the same day and within the same time window (13:00–16:00 local time) as visual canopy wilting assessments. Flights were conducted under clear to partially cloudy conditions to minimize illumination variability. In 2023 and 2024, multispectral imagery was acquired using a MicaSense RedEdge-M sensor (MicaSense Inc., Seattle, WA, USA) mounted on a multirotor UAV. The RedEdge-M sensor captures five discrete spectral bands, including blue (475 nm), green (560 nm), red (668 nm), red edge (717 nm), and near-infrared (840 nm), with a spatial resolution of 1280 × 960 pixels and a focal length of 6 mm. In 2025, UAV imagery was collected using an integrated multispectral camera onboard the SOTEN UAV (ACSL Inc., Japan). The SOTEN multispectral system captures three spectral bands, including green (560 nm), red (668 nm), and near-infrared (840 nm). The integrated sensor and camera system provided synchronized image acquisition without the use of external payloads. All UAV missions were planned and executed using the manufacturer-recommended flight planning software. Before each flight, images of manufacturer-provided reflectance panels with known properties were collected to standardize image brightness and support radiometric calibration. For the MicaSense system, flights were conducted at 36 m above ground level (AGL) with 75% forward overlap and 80% side overlap. For the SOTEN UAV, flights were carried out at 48 m AGL with 80% overlap in both forward and side directions. Across all years, a single UAV flight was conducted annually during the R4 to R5 growth stage in both irrigated and rainfed experimental fields.

### 2.4 UAV image data processing and trait extraction

Multispectral UAV imagery was processed using PIX4DMAPPER (v4.10.0; Pix4D SA, 2025) following a standard structure-from-motion workflow. Radiometric calibration was conducted using manufacturer-supplied reflectance panels to convert raw digital numbers into surface reflectance. Images were then aligned and bundle-adjusted to produce georeferenced, orthorectified orthomosaics (**Figure 1B**). These orthomosaics were then used for plot-level canopy trait extraction. All subsequent image analyses were carried out in R software (version 4.5.1) (R Core Team, 2025). Plot boundaries were delineated by generating a plot shapefile using the *FIELDImageR.Extra* R package (Pawar and Matias, 2023). To facilitate canopy–soil separation, the Normalized Difference Vegetation Index (NDVI) was calculated from the orthomosaics to help distinguish green plant canopy from bare soil. Otsu’s thresholding method (Otsu, 1979) was then applied to the NDVI layer to separate vegetation pixels from soil within each plot (**Figure 1C**). This method has been widely used to separate vegetation from bare soil in UAV−based phenotyping studies (Herzig et al., 2021; Marcial-Pablo et al., 2019). Pixels classified as soil were removed before further analysis. Vegetation indices (VIs) including Normalized Green Red Difference Index (NGRDI), Green Soil Adjusted Vegetation Index (GSAVI), Anthocyanin Reflectance Index (ARI), Chlorophyll Index – Red Edge (CIRE), Normalized Difference Vegetation Index (NDVI), Green Optimized Soil Adjusted Vegetation Index (GOSAVI), Chlorophyll Index-Green (CIG), Optimized Soil Adjusted Vegetation Index (OSAVI) and Green Normalized Difference Vegetation Index (GNDVI) were calculated from soil-masked orthomosaics using raster operations implemented in the *terra* R package (Hijmans, 2025) (**Figure 1D**). For plot-level analysis, mean vegetation index values were calculated by averaging pixel values within each plot using the *fieldInfo extra* function of the *FIELDImageR.Extra* package (Pawar and Matias, 2023). **Table 2** summarizes the vegetation indices and their calculation formulas used in this study. Imagery from each UAV flight was processed and analyzed independently at the plot level to enable consistent, objective phenotyping across treatments. Within each year, relationships between vegetation indices, visual wilting scores, and grain yield were quantified using these plot-level observations. For genotype-level cluster analysis, the three replicate plots per genotype were averaged to produce a single representative value for that year, allowing direct comparison between UAV-based metrics and visual assessments. No averaging was performed across years to maintain the independence of year-specific environmental effects.

**Table 2:** Vegetation indices evaluated for soybean water stress phenotyping.

Index	Formula	Reference	Physiological relevance
CIG	$\frac{NIR}{G}-1$	Gitelson et al. (2003)	Chlorophyll content
CIRE	$\frac{NIR}{RE}-1$	Gitelson et al. (2003)	Red-edge chlorophyll content
OSAVI	$\frac{NIR\;-R}{NIR+R+0.16}$	Rondeaux et al. (1996)	Soil-adjusted canopy greenness
GSAVI	$\frac{\text{NIR}-G}{NIR+G+0.5}\;\times \left(1+0.5\right)$	Sripada (2005)	Soil-adjusted canopy greenness
GNDVI	$\frac{NIR-G}{NIR+G}$	Gitelson et al. (1996)	Chlorophyll content and photosynthetic activity
GOSAVI	$\frac{NIR-G}{NIR+G+0.16}$	Rondeaux et al. (1996)	Soil-adjusted canopy greenness
NDVI	$\frac{NIR-R}{NIR+R}$	Rouse et al. (1974)	Chlorophyll, LAI, biomass, yield
NGRDI	$\frac{G-R}{G+R}$	Tucker (1979)	Canopy greenness
ARI	$\frac{1}{G}-\frac{1}{RE}$	Gitelson et al. (2001)	Anthocyanin content

Where *NIR*, *R*, *G*, and *RE* represent the near-infrared, red, green, and red-edge reflectance, respectively, acquired by the multispectral camera.

### 2.5 Correlation analysis between UAV−derived and agronomic traits

Pearson correlation analyses were performed at the plot level to evaluate relationships between UAV-derived vegetation indices and grain yield under both rainfed and irrigated conditions. Correlations were calculated separately for each condition (DT and IRR) and independently for each growing season (2023, 2024, and 2025) to account for year-specific environmental variability. Visual canopy wilting scores, recorded on a 1 (no wilting) to 5 (dead plant) scale, were included to enable direct comparison between traditional visual assessments and UAV-based phenotyping metrics. Statistical significance was assessed at (*p< 0.05*). To account for the influence of phenological development on UAV-derived vegetation indices, we quantified the extent to which maturity confounded relationships with yield. For each vegetation index, we computed its correlation with yield, its correlation with days to maturity, and the partial correlation with yield after statistically adjusting for maturity. This procedure ensured that drought-related canopy responses were interpreted independently of maturity effects.

Drought tolerance was evaluated using two yield-based indices derived from grain yield under rainfed (Yield*_DT_*) and irrigated (Yield*_IRR_*) conditions. The Relative Yield Index (RYI) and Geometric Mean Productivity (GMP) were applied to capture complementary aspects of genotype performance. RYI measures the extent to which a genotype maintains yield under drought relative to its performance under irrigated conditions, providing a direct indicator of yield retention (Fischer and Maurer, 1978) (Equation 1). In contrast, GMP summarizes performance across both stress and non-stress environments and therefore highlights genotypes that combine drought tolerance with strong yield potential (Fernandez, 1992). RYI and GMP were computed following the definitions proposed by Fischer and Maurer (1978) and Fernandez (1992), respectively (Equation 2).

(1) $$\text{RYI}=({\text{Yield}}_{DT}/{\text{Yield}}_{IRR})*100$$

(2) $$\text{GMP}=\sqrt{{\text{Yield}}_{DT}\times {\text{Yield}}_{IRR}}$$

### 2.6 Clustering analysis of UAV-derived vegetation indices under drought stress

To evaluate canopy responses under drought stress, *k*-means clustering (MacQueen, 1967) was applied to genotype-level mean vegetation indices derived from UAV imagery of rainfed plots. All vegetation indices were standardized (mean = 0, SD = 1) before clustering to ensure equal weighting and to avoid bias from differences in scale. Clustering was performed separately for each growing season to account for differences in weather and sensor configurations. Unsupervised *k*-means clustering was conducted in R using functions from the *stats* package (R Core Team, 2025). The optimal number of clusters (*k*) was evaluated using the elbow method, which examines the reduction in within-cluster sum of squares as a function of increasing *k* (Syakur et al., 2018). To visualize multivariate structure and aid interpretation of clustering patterns, Principal Component Analysis (PCA) was performed on the standardized vegetation indices, with analyses conducted independently for each growing season. Following clustering, grain yield, drought-response indices (RYI, and GMP), and visual wilting score were summarized at the genotype level for each cluster. Differences in yield and drought-response traits between clusters were assessed using Welch’s analysis of variance (ANOVA), as implemented in the *rstatix* R package (Kassambara, 2023). Welch’s ANOVA was selected because it does not assume equal variances or balanced group sizes, making it appropriate for comparisons involving algorithm-defined clusters with unequal membership. All statistical analyses and visualizations were performed using R (version 4.5.1; R Core Team, 2025).

## 3 Results

### 3.1 Variations in vegetation indices under irrigated and rainfed conditions across years (2023–2025)

**Figure 2:**
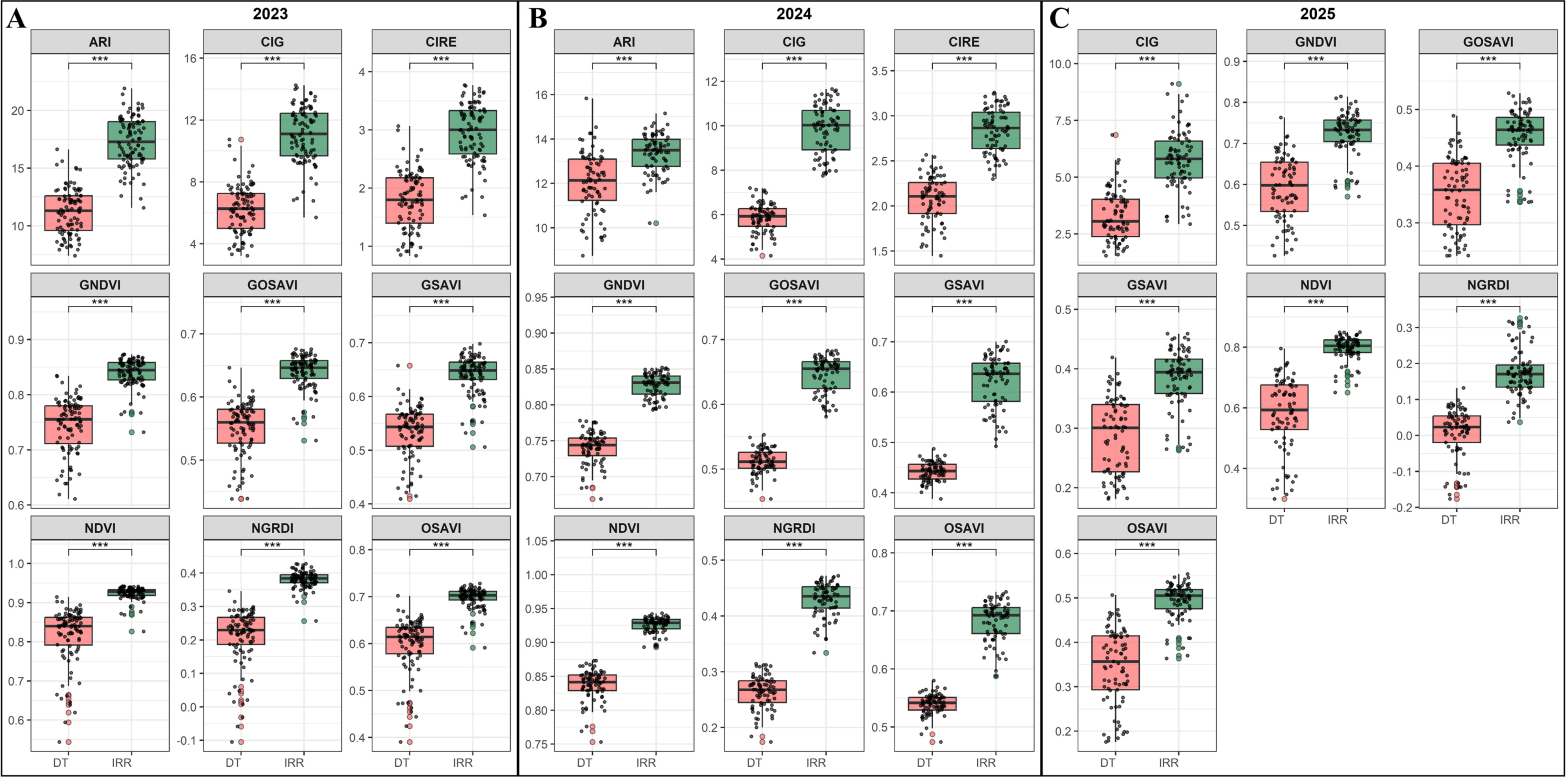
Comparison of vegetation indices under irrigated (IRR) and rainfed (DT) conditions across three growing seasons (2023–2025). Boxplots show the distributions of vegetation index values for the IRR (green) and DT (orange) treatments. (**A–C**) correspond to 2023, 2024, and 2025, respectively. Asterisks indicate significant differences between treatments (*p < 0.05, **p < 0.01, ***p < 0.001).

Vegetation indices consistently distinguished irrigated from rainfed soybean plots across all three study years (**Figure 2**). All nine indices showed highly significant treatment effects in 2023 and 2024 (p< 0.001), while seven remained significant in 2025 (p< 0.001). Across years, irrigated plots maintained higher median values for every index, reflecting greater canopy vigor under adequate water supply. Specifically, greenness metrics showed clear separation between treatments. NDVI ranged from 0.81 to 0.93 under irrigation compared with 0.59 to 0.84 under drought, while GNDVI ranged from 0.73 to 0.85 and 0.60 to 0.76, respectively. Chlorophyll-related indices (CIG, CIRE) exhibited the largest treatment differences, particularly CIG, which nearly doubled under irrigation in 2023 (11.1 vs. 6.3) and 2024 (10.0 vs. 5.9), and remained strongly separated in 2025 (5.8 vs. 3.5). Soil-adjusted indices confirmed the same pattern: GSAVI ranged from 0.39 to 0.64 under irrigation versus 0.29 to 0.53 under drought, OSAVI from 0.49 to 0.70 versus 0.35 to 0.60, and GOSAVI from 0.46 to 0.64 versus 0.35 to 0.55 across 2023-2025.

### 3.2 Variation in visual canopy wilting, grain yield and drought indices under rainfed conditions

**Figure 3:**
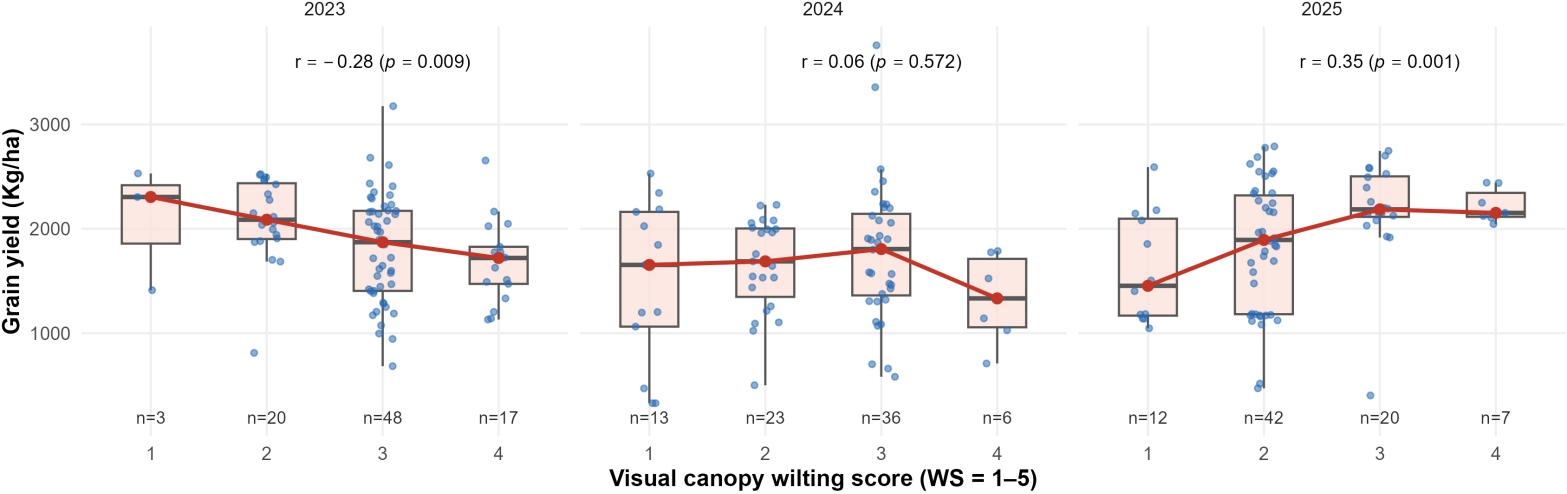
Relationship between grain yield and visual canopy wilting score (WS) under rainfed (DT) conditions across three growing seasons (2023–2025). Boxplots show the distribution of grain yield (kg/ha) for each wilting score category (WS = 1–5). Red points and connecting lines indicate median yield values across wilting score categories within each year. Panels represent individual years with a common y-axis scale. Pearson correlation coefficients (*r*) between yield and WS are shown within each panel, and sample sizes (*n*) for each wilting score category are displayed below the x-axis.

Wilting severity, grain yield, RYI, and GMP showed substantial variation among years under rainfed conditions (**Table 3**). In 2023, the evaluated genotypes exhibited a mean wilting score of 2.90 ± 0.74 (range 1–4), with grain yield averaging 1857.50 ± 490.93 kg ha^-^¹ and RYI of 42.56 ± 10.47%. The corresponding GMP was 2849.60 ± 562.46 kg ha^-^¹. In 2024, wilting severity averaged 2.45 ± 0.86, while grain yield and RYI were 1658.94 ± 622.68 kg ha^-^¹ and 37.98 ± 14.38%, respectively; GMP for this year averaged 2632.71 ± 640.27 kg ha^-^¹. In 2025, the genotype set evaluated showed a mean wilting score of 2.27 ± 0.82, grain yield of 1920.50 ± 574.16 kg ha^-^¹, and RYI of 53.43 ± 14.98%, with a GMP of 2620.58 ± 634.87 kg ha^-^¹. Within each year, wilting scores spanned a similar range (WS = 1 – 4), indicating the presence of contrasting levels of canopy stress among the evaluated genotypes (**Figure 3**). The distribution of grain yield within these wilting categories further illustrates the degree of within-season variability in yield response under drought conditions. Grain yield and RYI exhibited wide ranges in all years, reflecting considerable variability in yield performance and stress response among genotype sets evaluated in each season. Although mean values differed among years, these differences should be interpreted as year-specific distributions, as the genotype composition was not consistent across years.

**Table 3:** Summary statistics of wilting score, grain yield, RYI, and GMP under rainfed conditions.

Year	Wilting score	Yield (kg ha^-^¹)	RYI (%)	GMP (kg ha^-^¹)
2023	2.90 ± 0.74 (1– 4)	1857.50 ± 490.93 (684.01 – 3175.50)	42.56 ± 10.47 (22.37 – 78.74)	2849.60 ± 562.46 (1447.04 – 4092.40)
2024	2.45 ± 0.86 (1– 4)	1658.94 ± 622.68 (329.13 – 3758.50)	37.98 ± 14.38 (6.77 – 75.82)	2632.71 ± 640.27 (1160.58 – 4327.73)
2025	2.27 ± 0.82 (1– 4)	1920.50 ± 574.16 (403.37 – 2789.56)	53.43 ± 14.98 (15.22 – 86.89)	2620.58 ± 634.87 (1021.39 – 3705.86)

Values represent mean ± standard deviation, with minimum–maximum shown in parentheses.

### 3.3 Relationships between UAV−derived vegetation indices, visual wilting score and yield-related traits

**Figure 4:**
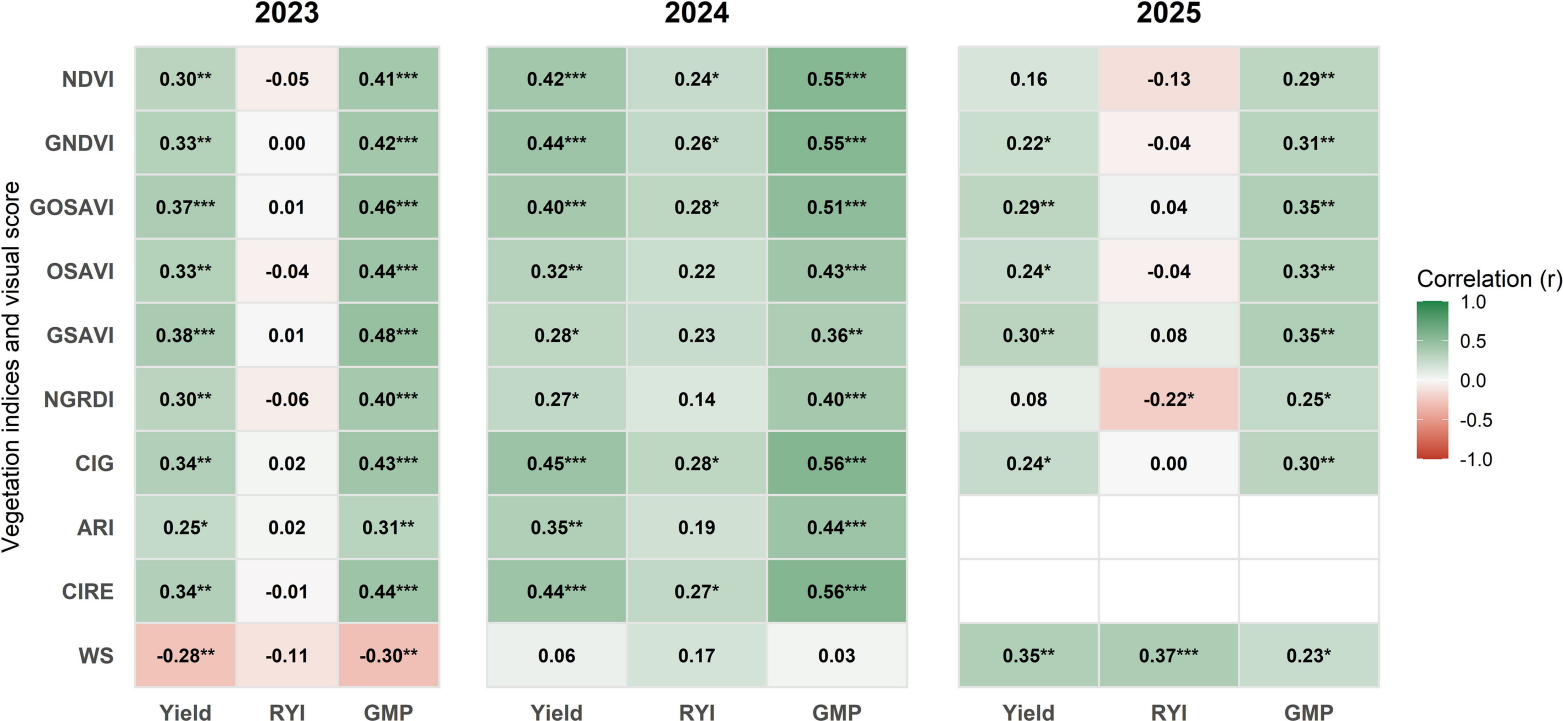
Heat maps showing Pearson correlation coefficients between UAV-derived vegetation indices, WS, and yield-related traits under rainfed conditions across the 2023–2025 growing seasons. Columns represent grain yield, RYI, and GMP, while rows represent individual vegetation indices and visual wilting score. Each panel corresponds to a single growing season. Cell colors indicate the strength and direction of correlations (red = negative, green = positive), and numeric values denote correlation coefficients, with asterisks indicating statistical significance (*p* < 0.05, *p* < 0.01, *p* < 0.001). ARI and CIRE were not available in 2025 and are shown as blank.

The relationship between visual canopy wilting score and grain yield was inconsistent across years (**Figure 4**). In 2023, greater wilting severity was associated with reduced yield (*r* = −0.28, *p* = 0.009), and WS also showed negative associations with RYI (*r* = −0.11, not significant) and GMP (*r* = −0.30, *p* < 0.01). In 2024, WS exhibited no significant association with yield (*r* = 0.06), RYI (*r* = 0.17), and GMP (*r* = 0.03). Whereas, in 2025, WS was positively associated with yield (*r* = 0.35, *p* < 0.001), RYI (*r* = 0.37, *p* < 0.001), and GMP (*r* = 0.23, *p* < 0.05). The direction and strength of WS correlations with yield and drought indices varied among seasons, indicating that visual canopy wilting does not consistently reflect yield performance or drought tolerance under rainfed conditions. In contrast, UAV-derived VIs exhibited consistent and yield-relevant relationships. In 2023, VIs correlations with yield ranged from *r* = 0.25 – 0.38 (*p<* 0.05), whereas correlations with GMP were consistently higher (*r* = 0.40 – 0.48, *p<* 0.001). However, relationships with RYI were non-significant. In 2024, yield correlations ranged from *r* = 0.28 – 0.45 (*p<* 0.05), with several indices also showing significant but weaker associations with RYI (*r* = 0.24 – 0.28, *p<* 0.05), whereas correlations with GMP remained strongest (*r* = 0.36 – 0.56, *p<* 0.001). In 2025, correlations with yield were generally weaker (*r* = 0.16 – 0.30, *p<* 0.05) and RYI relationships were limited; however, most vegetation indices retained positive and significant associations with GMP (*r*= 0.25 – 0.35, *p<* 0.05). Among all traits, GMP exhibited the significant and most stable associations with vegetation indices every year. It indicates that, under rainfed conditions, VIs measured at the R4/R5 stage captured canopy responses to drought more effectively than visual wilting scores and showed a significant, consistent association with grain yield. Greenness-based indices, including NDVI, GNDVI, GOSAVI, and GSAVI, consistently showed significant associations with yield and stress indices. ARI and CIRE were not available in 2025 and were therefore excluded. A similar pattern was observed under irrigated conditions, where greenness, chlorophyll, and soil-adjusted indices (NDVI, GNDVI, CIG, GOSAVI, OSAVI, GSAVI) showed moderate to strong correlations with yield (*r* = 0.28 – 0.73, *p* < 0.01; **Supplementary Figure 2**). Pearson correlation analyses revealed strong interrelationships among UAV-derived vegetation indices under both irrigated and rainfed conditions, indicating high multicollinearity among the indices. This pattern likely reflects that, in both environments, indices constructed from overlapping spectral regions (for example, red–NIR or red-edge–NIR combinations) respond in a similar way to shared gradients in canopy traits, resulting in the high inter-index correlations observed. Despite this interdependence, the VIs consistently captured key aspects of canopy status, including vigor, greenness, and stress responses.

Across three growing seasons, vegetation indices showed consistent relationships with yield, with the strength of these associations varying after maturity adjustment (**Supplementary Figures S3**-**S5**). Several indices, including CIG, GOSAVI, and GNDVI, retained positive maturity-adjusted correlations with yield, indicating that these metrics captured physiological drought responses beyond phenological differences. Overall, the maturity-corrected results show that UAV-derived indices retain meaningful drought-related information even after maturity effects are adjusted.

### 3.4 Clustering of UAV-derived vegetation indices

**Figure 5:**
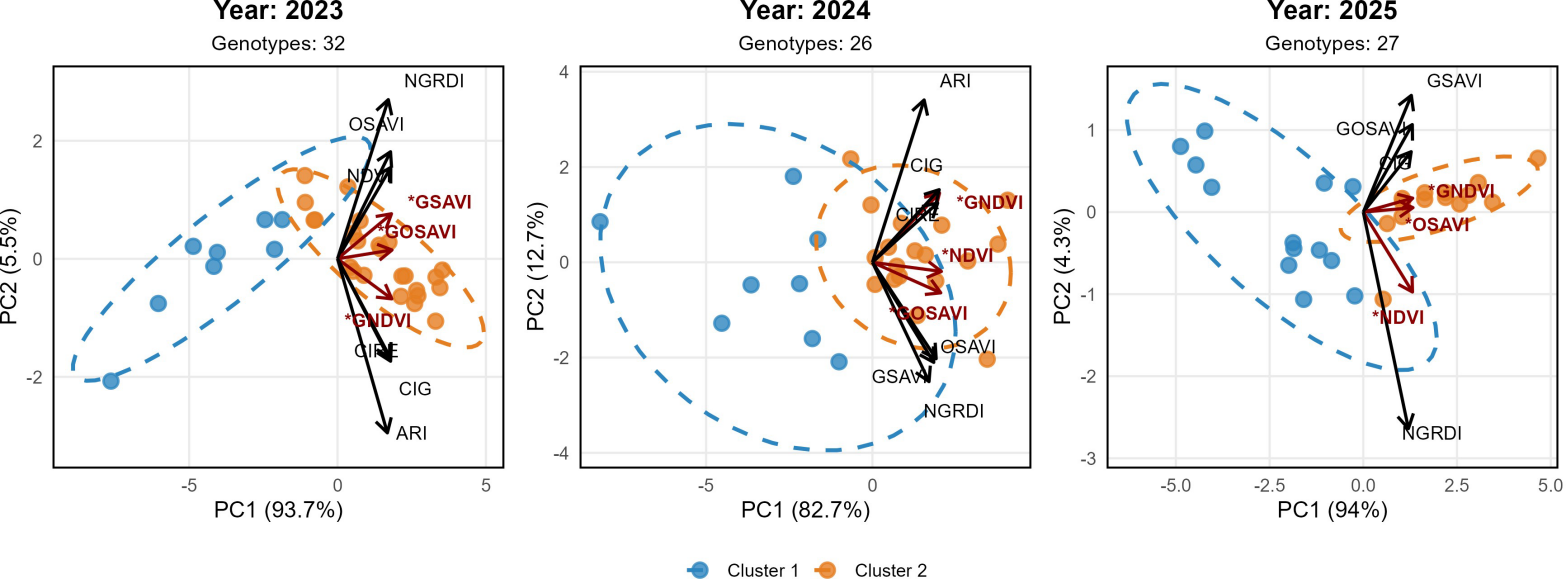
Principal Component Analysis (PCA) of UAV-derived vegetation indices for soybean genotypes under rainfed (DT) conditions across 2023–2025. Points represent individual genotypes, color-coded by cluster assignment: Cluster 1 (blue) and Cluster 2 (orange), identified using k-means clustering (n = 2). Shaded areas indicate 95% confidence ellipses for each cluster. Arrows represent index loadings on PC1 and PC2; the three indices with the largest absolute loadings on PC1 are highlighted with colored, bold labels.

Elbow method analysis conducted independently for each growing season showed a consistent reduction in within−cluster sum of squares at *k*= 2, with only marginal decreases observed for higher cluster numbers (**Supplementary Figure S6**). This pattern indicated that two clusters captured the dominant structure of variation in UAV−derived vegetation indices across years. The first two principal components explained most of the variance, accounting for 98.8% in 2023, 91.4% in 2024, and 97.4% in 2025 (**Figure 5**). PC1 alone captured 82.7–94.0% of total variation, indicating strong collinearity among indices and a shared physiological signal associated with drought response. PCA loading vectors identified GNDVI as a major contributor to cluster differentiation in all years. Additional influential indices included GOSAVI and NDVI in 2023, GOSAVI and GSAVI in 2024, and OSAVI and NDVI in 2025 (**Figure 5**). Although there was limited overlapping of genotypes across years, the UAV-derived vegetation indices showed similar patterns each season, producing comparable groupings of genotypes.

### 3.5 Comparative performance of UAV−based phenotyping and visual wilting

Unsupervised *k*-means clustering (*k* = 2) based on UAV-derived vegetation indices separated genotypes into groups with consistent differences in yield performance across the 2023–2025 rainfed seasons (**Table 4**). In 2023, grain yield (1,925.8 vs. 1,703.1 kg ha^-^¹) and RYI (42 vs. 45 %) did not differ significantly between clusters. In contrast, Cluster 2 showed significantly higher GMP than Cluster 1 (GMP: 2,951.0 vs. 2,590.7 kg/ha, *p* < 0.05), accompanied by a marginal but significant reduction in wilting score (2.8 vs. 3.1). The separation increased in 2024, when Cluster 2 produced 50% higher yield (1,849.9 *vs.* 1,229.2 kg/ha) and greater GMP (2,887.0 *vs.* 2,096.1 kg/ha), while wilting scores remained nearly identical between groups (2.5 *vs.* 2.4). RYI values also reflected this difference in yield retention (41% *vs.* 33%). In 2025, grain yield (2,056.7 vs. 1,773.8 kg ha^-^¹), GMP (2,783.0 vs. 2,441.0 kg ha^-^¹), RYI (55 vs. 52 %), and wilting score (2.5 vs. 2.0) did not differ significantly between Cluster 1 and Cluster 2, although Cluster 2 showed numerically higher yield-related traits and a lower mean wilting score.

**Table 4:** Cluster-specific agronomic performance of soybean genotypes under rainfed conditions (2023-2025).

Year	Trait	Cluster 1	Cluster 2
2023	Yield (kg/ha)	1,703.1^a^	1,925.8^a^
	GMP (kg/ha)	2,590.7^a^	2,951.0^b^
	RYI (%)	45^a^	42^a^
	WS (1–5)	3.1^a^	2.8^b^
	Genotypes	8	24
2024	Yield (kg/ha)	1,229.2^a^	1,849.9^b^
	GMP (kg/ha)	2096.1^a^	2887.0^b^
	RYI (%)	33^a^	41^b^
	WS (1–5)	2.5^a^	2.4^a^
	Genotypes	8	18
2025	Yield (kg/ha)	1,773.8^a^	2,056.7^a^
	GMP (kg/ha)	2,441.0^a^	2,783.0^a^
	RYI (%)	52^a^	55^a^
	WS (1–5)	2.5^a^	2.0^a^
	Genotypes	13	14

Different superscript letters within rows indicate significant differences between clusters based on Welch’s ANOVA (p< 0.05). GMP, Geometric Mean Productivity RYI, Relative Yield Index; WS, Wilting Score (1–5 scale).

Across years, clustering based on UAV-derived vegetation indices differentiated genotypes primarily with respect to yield performance, particularly GMP, under rainfed conditions, although the strength of separation varied among years. In contrast, clustering based on WS alone captures variation in visible canopy stress but does not reliably differentiate genotypes by yield performance under rainfed conditions (**Supplementary Table 2**).

**Figure 6:**
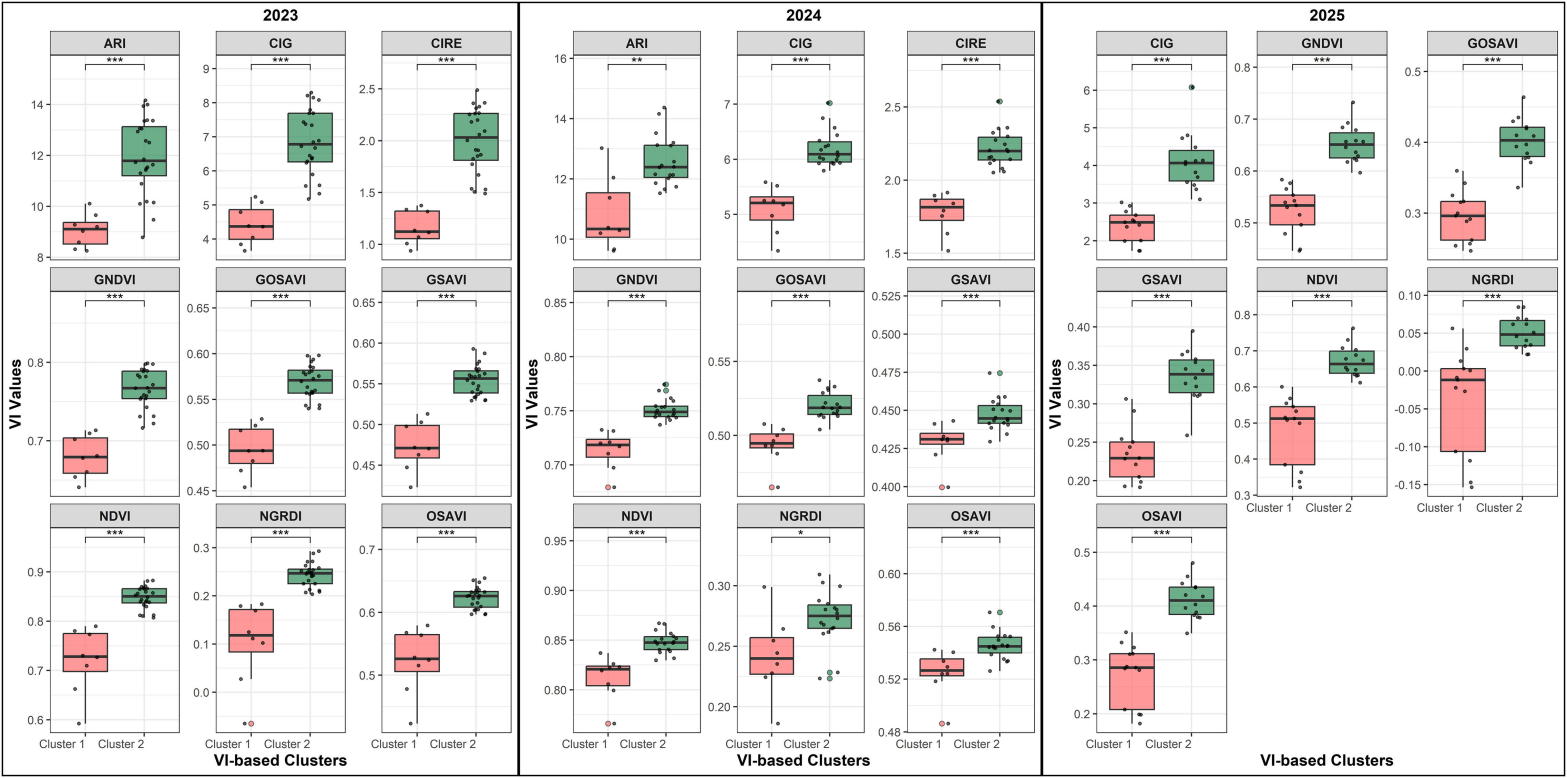
Boxplots of vegetation indices (genotypic averages) for rainfed soybean plots, grouped into two clusters based on their vegetation index patterns across 2023–2025. Cluster 1 (red) and Cluster 2 (green) represent distinct phenotypes. Asterisks indicate significant differences between clusters (*p < 0.05, **p < 0.01, ***p < 0.001).

These clustering outcomes were further supported by visualization of individual indices, as most vegetation indices (NDVI, GNDVI, CIG, GOSAVI, OSAVI, GSAVI, CIRE) showed consistent and significant differences between clusters across seasons (**Figure 6**). Compared with visual canopy wilting scores, which rely on subjective assessment, clustering based on UAV-derived indices provides a quantitative, high-throughput approach. This framework supports the identification of genotypes with higher yield potential and consistent productivity under drought. Indices such as GNDVI, which contributed strongly across seasons, appear particularly informative for detecting canopy responses associated with yield retention. These results indicate that UAV-based phenotyping captures yield-relevant physiological variation that is often missed by visual assessments.

## 4 Discussion

In crop breeding, drought tolerance is defined as the genetic ability of a genotype to produce significantly higher yield than drought-susceptible genotypes under drought stress (Fleury et al., 2010; Sallam et al., 2019; Nguyen et al., 2024). It is therefore determined primarily by yield performance under stress, rather than merely by physiological appearance during stress. Drought tolerance in crops is a complex quantitative trait because plants respond to water stress in many ways, from visible morphological changes to molecular adjustments, throughout all stages of growth (Farooq et al., 2009). In conventional drought-tolerant soybean breeding, yield under irrigated and rainfed conditions is the most important genetic trait to evaluate soybean drought tolerance. In conventional soybean breeding programs, visual canopy wilting has been widely used as a practical proxy for drought response (Manjarrez-Sandoval et al., 2020; Fallen et al., 2023; Wu et al., 2025). However, canopy wilting is inherently subjective, environmentally sensitive, and typically measured at a single time point, which limits its reliability. Studies have shown that wilting scores correlate weakly and inconsistently with yield across environments (Ye et al., 2019; Bellaloui et al., 2026). In addition, canopy wilting exhibits moderate heritability and is a polygenic trait governed by environmentally sensitive QTLs (Charlson et al., 2009). Consistent with this, Saleem et al. (2022) reported that canopy wilting scores are highly sensitive to short-term micro-climate conditions, showing inconsistent patterns over time with low-to-moderate heritability (H^2^ = 0.16 – 0.51). In our multi-year rainfed trials, wilting scores were likewise not consistently associated with grain yield. These findings reinforce that drought tolerance assessed by wilting scores is not meaningful unless it translates into improved yield retention under drought stress. Therefore, breeding programs should focus on identifying genotypes that maintain yield potential and retention under rainfed conditions rather than relying on leaf turgor as a proxy for drought resilience. UAV-based multispectral phenotyping offers a quantitative, scalable, and objective alternative to visual wilting assessments. Vegetation indices derived from multispectral sensors capture canopy vigor, greenness, and physiological status in ways that are closely linked to yield formation under rainfed conditions (Maimaitijiang et al., 2020; Zhou et al., 2020; Tahery et al., 2025). However, the strength of relationships between UAV-based VIs and yield is influenced by environmental conditions. Stronger correlations between vegetation indices and yield under irrigated conditions likely arise because well-watered crops maintain uniform canopy structure and stable chlorophyll and biomass distribution, leading to more consistent reflectance signals that track productivity (Hatfield and Prueger, 2010). In contrast, drought introduces variability in plant water status, leaf structure, and canopy condition, which alters reflectance patterns and can weaken vegetation index–yield relationships under stress (Gutierrez et al., 2010). In our three−season dataset, genotypes grouped by UAV−derived VIs (greenness/chlorophyll and soil−adjusted indices) showed consistent differences in yield, GMP, and RYI even when canopy wilting scores were similar, indicating that VIs captured yield−relevant canopy physiology that CWS missed. Stronger correlations between vegetation indices and GMP than RYI are expected, as VIs capture integrated canopy productivity traits that align with absolute yield performance across environments, whereas RYI is ratio-based and more sensitive to environmental noise, a pattern supported by UAV-based drought evaluations using GMP (Liang et al., 2024). Additionally, vegetation indices measured during R4 to R5 under rainfed conditions showed consistent, positive correlations with yield (*r* = 0.24 – 0.45), whereas CWS did not. Several indices repeatedly contributed to genotype separation, including GNDVI, NDVI, and soil-adjusted greenness metrics (GSAVI, GOSAVI, OSAVI). This is consistent with evidence from the study by Zhou et al. (2020), which shows that indices such as NDVI and GNDVI correlate strongly with yield under water-limited conditions. Zhou et al. (2020) also reported that these indices exhibit higher heritability (NDVI = 0.67; GNDVI = 0.81) than manual wilting scores (H^2^ = 0.58), supporting their repeatability and usefulness in breeding pipelines. In our trials, multispectral information captured during R4 to R5 diverged among genotypes, enabling earlier classification of drought response and yield retention under rainfed conditions. This early signal aligns with studies that red−edge and near−infrared bands enable pre−visual detection of water−limiting stress before visual symptoms appear, enabling earlier and more informed selection decisions (Jones et al., 2024; Liang et al., 2024; Tavares et al., 2022). In our dataset, vegetation indices grouped genotypes in patterns that were generally consistent with yield performance and highlighted some high-yielding, drought-tolerant lines that did not necessarily show low wilting scores. This demonstrates that UAV-based phenotyping captures genetic variation relevant to yield retention that visual wilting fails to detect. Overall, UAV imaging supports the transition from subjective drought screening toward a more objective, high-throughput, and data-driven process that aligns with modern breeding goals.

The integration of UAV-derived spectral metrics into breeding programs has important implications for accelerating genetic gain under drought. These quantitative metrics enable the identification of drought-tolerant genetic resources based on yield performance rather than wilting symptoms alone. They support early-stage and large-scale selection, allowing breeders to evaluate thousands of plots objectively and consistently. Collectively, these advantages support a shift toward yield-centered drought-tolerance breeding, where UAV phenotyping becomes a core tool for identifying superior genetic materials under rainfed conditions.

Despite the promise of UAV multispectral phenotyping, several limitations of this study should be acknowledged. Genotype overlaps across years were limited, reducing the ability to track genetic performance longitudinally. Conducting the study at a single location restricted environmental inference and limited the evaluation of genotype × environment interactions. UAV data collection was limited to a single flight per season during the R4 to R5 stage, preventing analysis of temporal drought progression, canopy recovery, and season-long stress dynamics. Additionally, environmental variability across years introduced uncontrolled factors that may have influenced vegetation index performance. Future work should incorporate multi-temporal UAV flights across key growth stages, larger and more consistent genotype panels, multi-location trials to capture environmental diversity, and integration of spectral data with physiological and genomic datasets. These improvements will enhance the robustness, interpretability, and generalizability of UAV-based drought-tolerance phenotyping.

## 5 Conclusion

In conclusion, this study demonstrates that UAV-derived vegetation indices provide consistent, objective, and yield-relevant indicators of drought stress in soybeans across multiple environments and growing seasons. Overall, this study indicates that UAV-derived vegetation indices are more consistently associated with yield performance under rainfed conditions than visual wilting scores. While visual assessments showed limited and variable relationships with yield-related traits, vegetation indices captured canopy responses closely linked to productivity. These findings highlight the value of UAV-based indices as objective, canopy-level indicators for drought assessment. UAV phenotyping, therefore, offers a scalable and less subjective complement to conventional drought screening, with the potential to support more efficient drought assessment in soybean breeding programs targeting water-limited environments.

## Data Availability

The raw data supporting the conclusions of this article will be made available by the authors, without undue reservation.
