# Supplementary material for: UAV-based phenotyping outperforms visual canopy wilting for evaluating soybean drought tolerance and yield retention under rainfed conditions
Source: Front Plant Sci. 2026 Jun 2;17:1835549. doi: 10.3389/fpls.2026.1835549 (PMC13269238; doi:10.3389/fpls.2026.1835549)
Supplement: Supplementary file 1 [file DataSheet1.docx]

**Supplementary Figures**


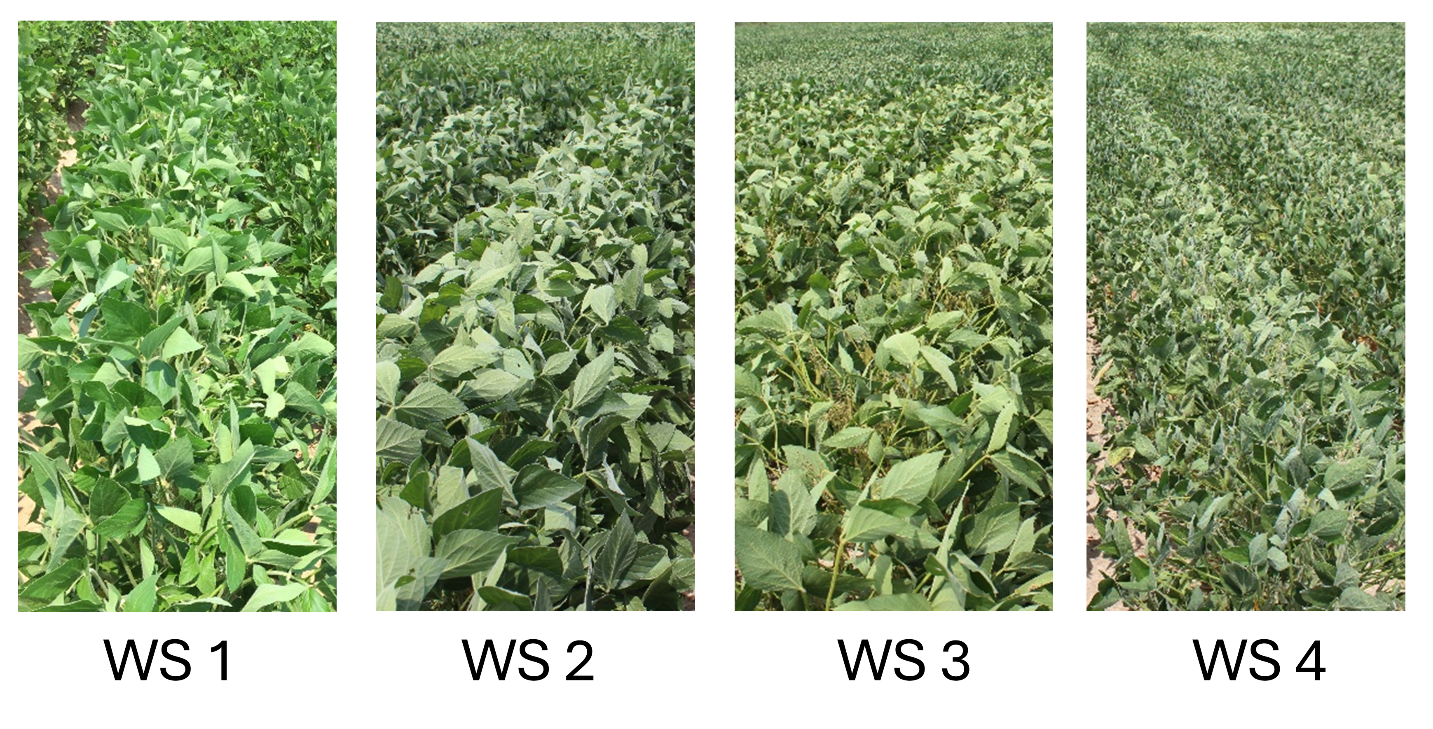


**Figure S1.** Representative canopy images illustrating visual wilting score levels (WS: 1–4) observed across the experimental seasons. Wilting score level 5 (partial plant death) was not observed during the study period.


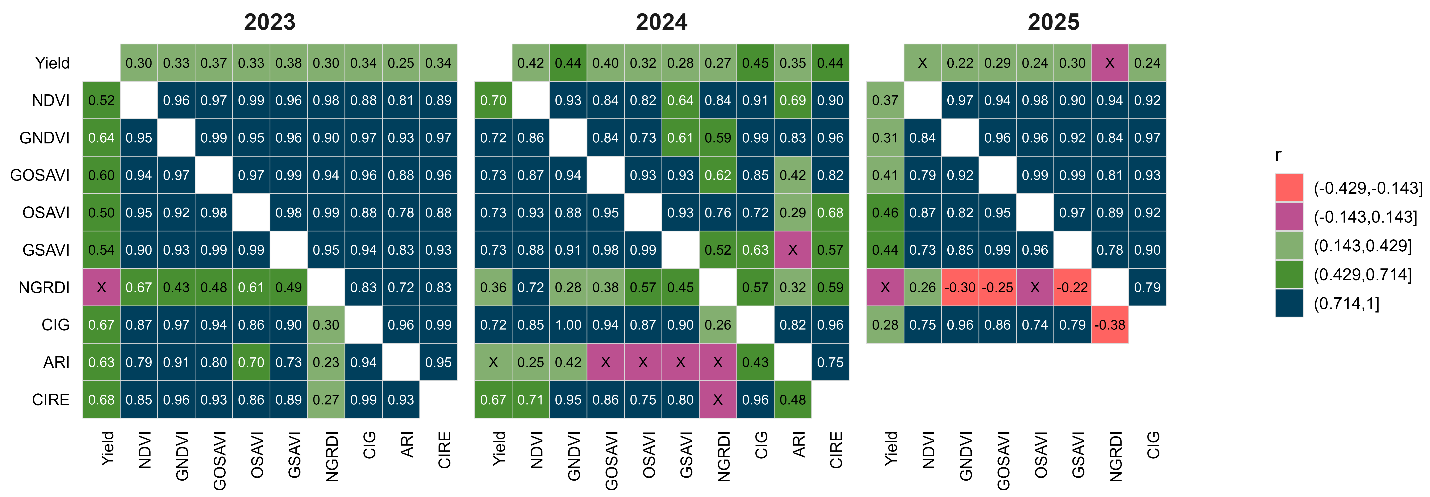


**Supplementary Figure S2.** Trait correlation analysis across three growing seasons (2023–2025). In each correlation matrix, the lower triangle shows Pearson correlations under irrigated (IRR) conditions, and the upper triangle shows correlations under rainfed (DT) conditions. Statistical significance of correlations was assessed using Pearson’s correlation test at α = 0.05. Non-significant correlations (*p* > 0.05) are indicated by “X”.


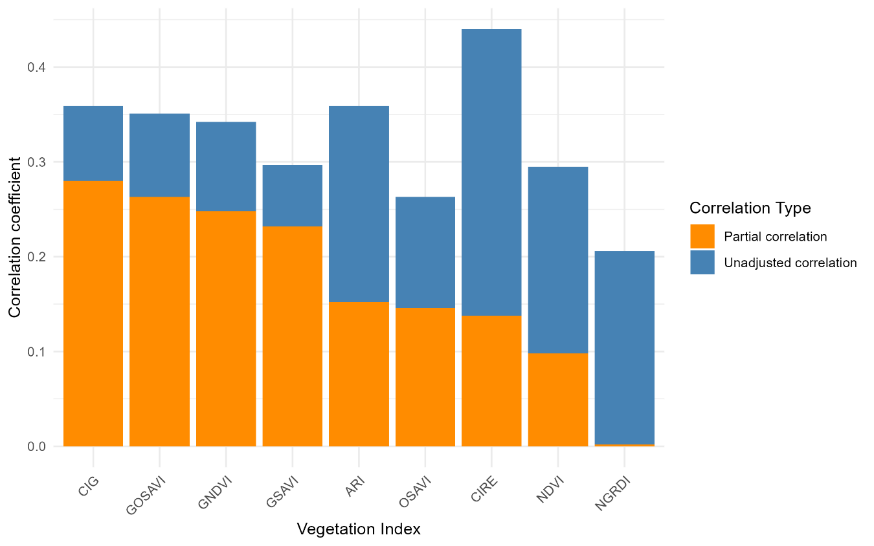


**Supplementary Figure S3.** Comparison of unadjusted correlations (index vs. yield) and maturity‑adjusted partial correlations for all UAV‑derived vegetation indices. Bars show how each index’s association with yield changes after accounting for phenological development (days to maturity). Higher partial correlations indicate indices that retain meaningful drought‑response information independent of maturity effects.


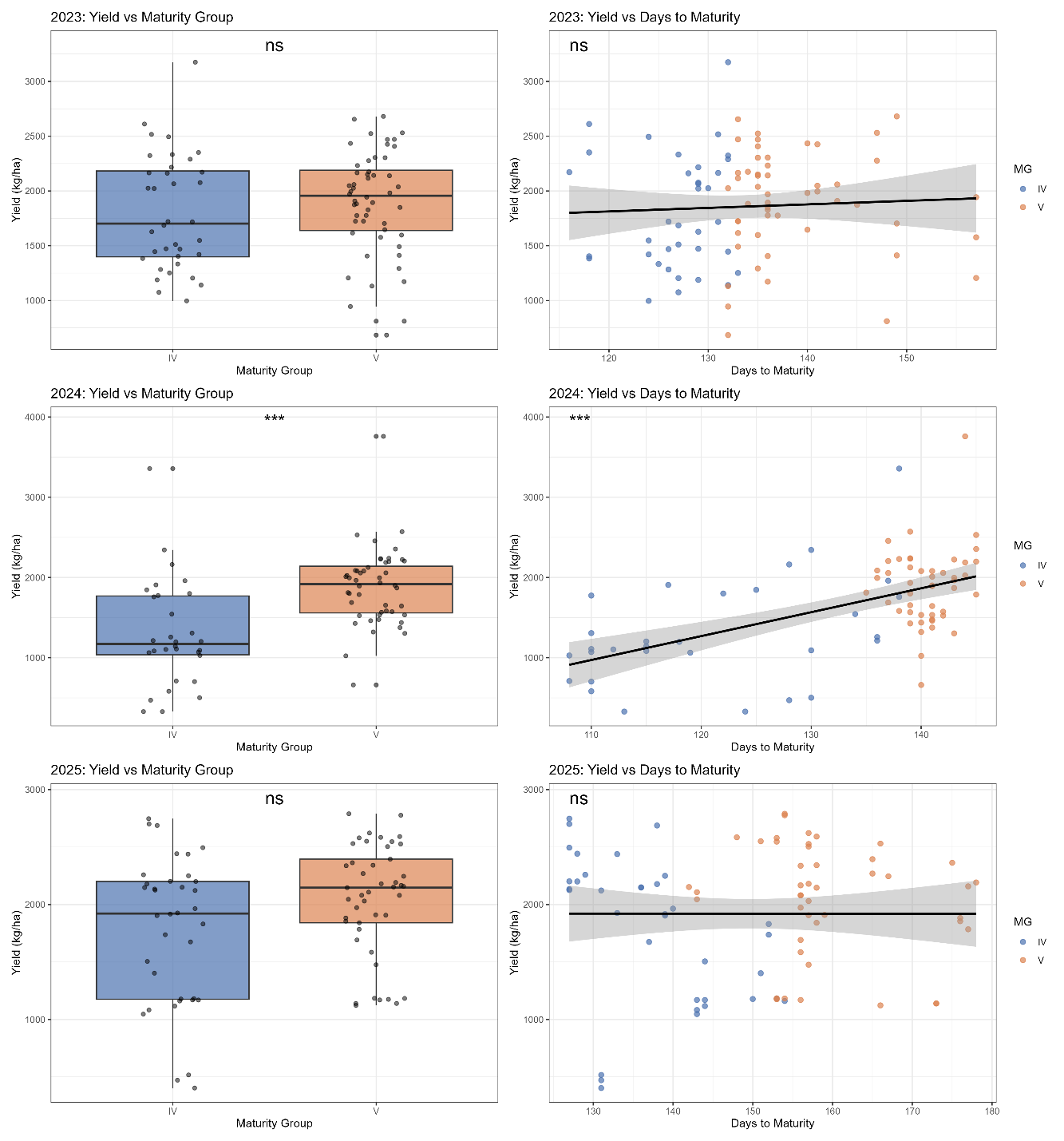


1. (B)

**Figure S4.** Yield responses across maturity groups (MG) and days to maturity (DTM) for 2023–2025. **Panel A** shows boxplots of yield by maturity group with Wilcoxon test significance indicated (*p* < 0.05 = *, <0.01 = **, <0.001 = ***). **Panel B** shows scatterplots of yield versus days to maturity with linear regression lines and correlation significance. Colors indicate maturity groups (IV = blue, V = orange). This visualization highlights both the effect of maturity group and continuous phenology on yield across years.


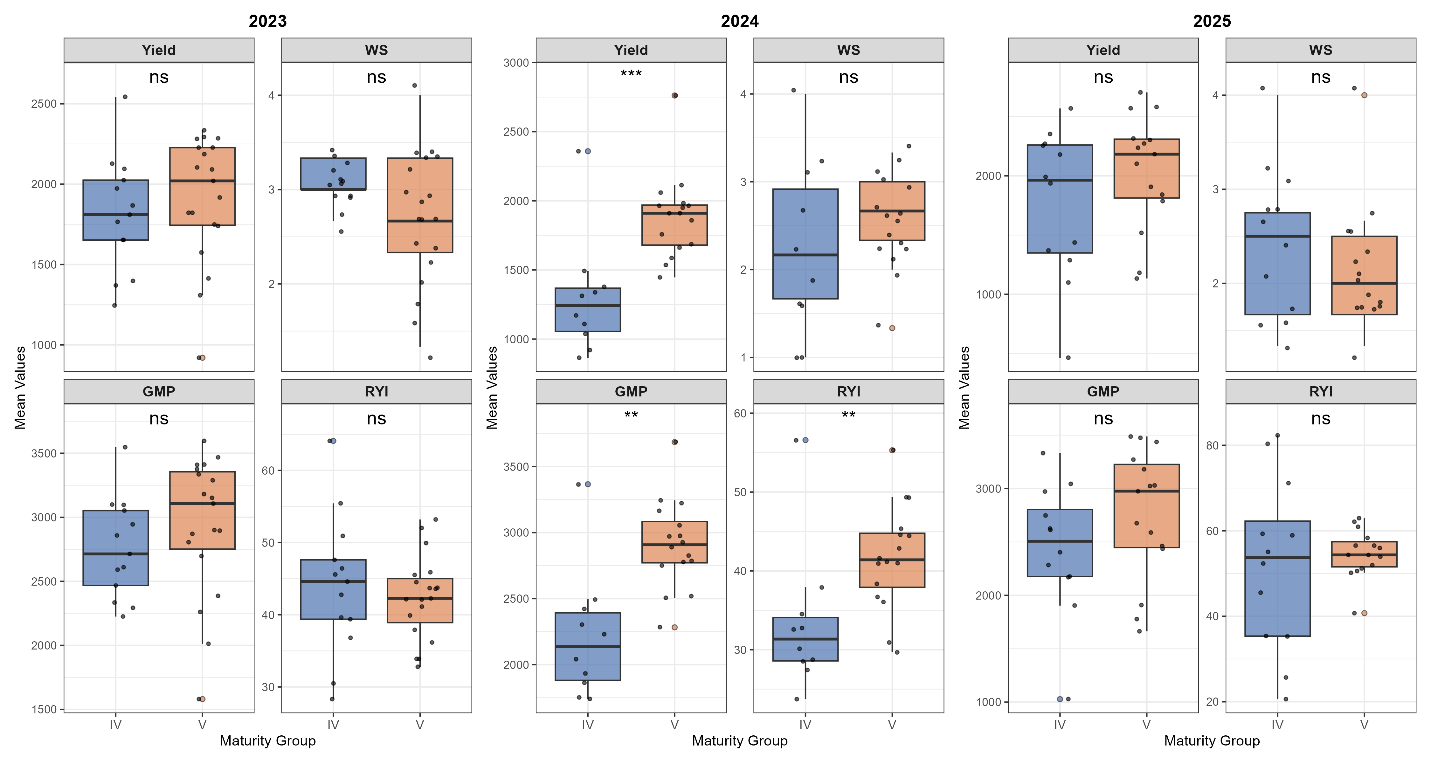


**Figure S5.** Mean UAV-derived vegetation index values across maturity groups (MG IV and V) for each soybean line over three growing seasons (2023–2025). Boxplots show the distribution of values per maturity group, with Wilcoxon test significance indicated above each index (*p* < 0.05 = *, <0.01 = **, <0.001 = ***). This visualization highlights how vegetation indices vary with maturity group across years, providing context for interpreting drought-related physiological responses.


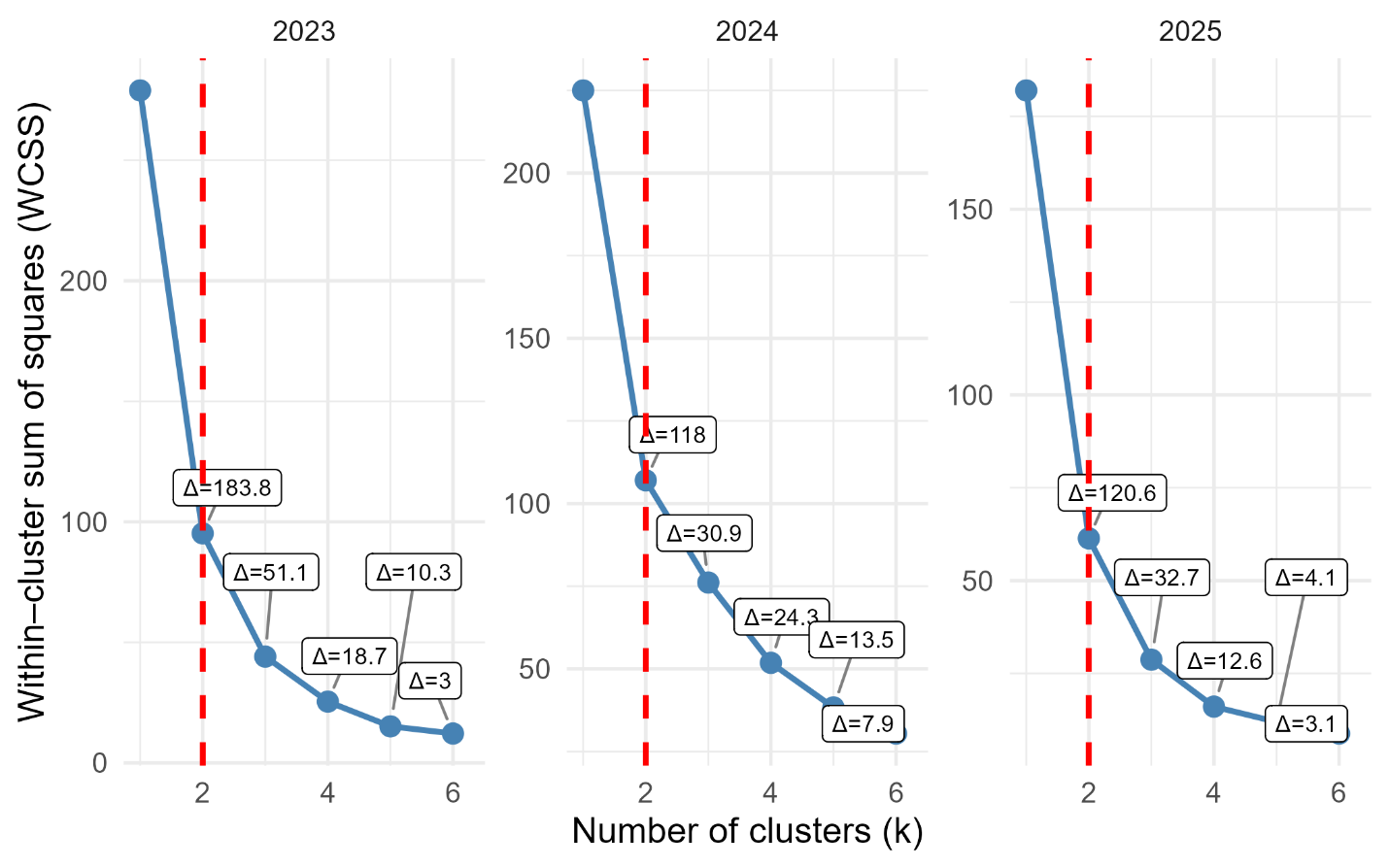


**Figure S6.** Elbow plots illustrating within‑cluster sum of squares (WCSS) for *k*‑means clustering based on UAV‑derived vegetation indices across the 2023–2025 rainfed seasons. ΔWCSS values quantify the reduction in within‑cluster variance associated with successive cluster increments (*k* − 1 → *6*). In all years, the largest ΔWCSS occurs at *k* = 2 (dashed line), supporting selection of two clusters.
