# Supplementary material for: UAV-based phenotyping outperforms visual canopy wilting for evaluating soybean drought tolerance and yield retention under rainfed conditions
Source: Front Plant Sci. 2026 Jun 2;17:1835549. doi: 10.3389/fpls.2026.1835549 (PMC13269238; doi:10.3389/fpls.2026.1835549)
Supplement: Supplementary file 3 [file Table2.docx]

**Table S2:** Comparison of grain yield, drought response indices, and visual wilting score between clusters derived from wilting‑score‑based *k*‑means clustering under rainfed conditions across the 2023–2025 growing seasons. Values represent cluster means. Clusters are defined as high wilting score (Cluster 1) and low wilting score (Cluster 2) based on *k*‑means clustering of mean WS values. Different superscript letters within a row indicate significant differences based on Welch’s ANOVA (p < 0.05). RYI = relative yield index; GMP = geometric mean productivity; WS = wilting score (1–5 scale).

| Year | Trait | Cluster1: High WS | Cluster2: Low WS |
| --- | --- | --- | --- |
| 2023 | Yield (kg/ha) | 1815.75^a^ | 2071.30^a^ |
|  | GMP (kg/ha) | 2784.15^a^ | 3154.02^a^ |
|  | RYI (%) | 43.1^a^ | 42.7^a^ |
|  | WS (1–5) | **3.1^a^** | **2.0^b^** |
|  | Genotypes | 25 | 7 |
| 2024 | Yield (kg/ha) | 1694.7^a^ | 1573.65^a^ |
|  | GMP (kg/ha) | 2663.1^a^ | 2602.57^a^ |
|  | RYI (%) | 40.3^a^ | 34.5^a^ |
|  | WS (1–5) | **2.8^a^** | **1.6^b^** |
|  | Genotypes | 18 | 8 |
| 2025 | Yield (kg/ha) | 2118.37^a^ | 1748.25^a^ |
|  | GMP (kg/ha) | 2763.97^a^ | 2488.25^a^ |
|  | RYI (%) | 58.0^a^ | 49.1^a^ |
|  | WS (1–5) | **2.9^a^** | **1.7^b^** |
|  | Genotypes | 13 | 14 |
